# Second Language Learners’ Competence of and Beliefs About Pragmatic Comprehension: Insights From the Chinese EFL Context

**DOI:** 10.3389/fpsyg.2021.801315

**Published:** 2022-01-07

**Authors:** He Yang

**Affiliations:** College of Foreign Languages and Cultures, Xiamen University, Xiamen, China

**Keywords:** learner beliefs, Ajzen’s theory of planned behavior, self-efficacy, learning behavior, pragmatic comprehension, conversational implicature

## Abstract

It can be a great challenge for second language (L2) learners to comprehend meanings that are implied in utterances rather than the surface meaning of what was said. Moreover, L2 learners’ attitudes toward pragmatic learning are unknown. This mixed-methods study investigates L2 learners’ ability to comprehend conversational implicatures. It also explores their beliefs about and intentions to develop this ability using Ajzen’s theory of planned behavior (TPB). A total of 498 freshmen from a public university in China participated in the study. Data were collected using a web-based test, stimulated recall tasks and semi-structured interviews. Results show that the participants differed in recognizing the intended meanings. Complicated factors account for the variations. In addition to the types of implicature, learners’ beliefs about developing pragmatic comprehension also influence their learning intention, and subsequent performance. These beliefs include learners’ multi-layered, complex attitudes toward the outcomes of pragmatic learning, perceived self-efficacy beliefs regarding language proficiency and L2 cultural knowledge, actual behavioral control over opportunities and resources for pragmatic learning, and perceptions of less social pressure on pragmatic learning. The use of TPB may help language teachers and test designers to understand learners’ beliefs about L2 pragmatic learning in the English as a foreign language (EFL) context. Understanding the factors influencing learners’ intention will help design more effective teaching curricula that may integrate pragmatic instruction and testing in the future.

## Introduction

Most learners, particularly adult learners, have certain beliefs about what is worth learning, how the instruction should be delivered and why they are devoted to certain learning activity (Dörnyei and Ushioda, 2013; Lightbown and Spada, 2013). Understanding these beliefs helps to account for learners’ learning motivation behavior (Alhamami, 2018). Beliefs have the potential to influence learners, their learning experience and their actions by either enhancing or interfering with their language learning (Alanen, 2003). In the era of globalization, acquisition of pragmatic competence is regarded as an indispensable aspect of second language (L2) learning (Taguchi and Ishihara, 2018). However, the roles of language learners’ beliefs about pragmatic competence and its acquisition have been largely under-explored. To date, with the exceptions of Yang and Ren (2019) and García-Gómez (2020), few studies have investigated the roles of learners’ internal characteristics, such as beliefs and motivation, in L2 pragmatic learning.

Pragmatic competence refers to the ability to use language accurately and appropriately in social interactions, including both productive and receptive pragmatic competences (Kasper and Rose, 2002; Ren, 2015). In the past decades, pragmatic competence has been analyzed mainly through production skills, especially performance of speech acts (e.g., Achiba, 2002; Felix-Brasdefer, 2004; Ren, 2013; Kurtyka, 2019), although an increasing number of studies have investigated learners’ ability to comprehend conventional expressions (Bardovi-Harlig and Bastos, 2011; Roever, 2012), conversational implicature (Taguchi, 2011; Taguchi and Bell, 2020; Ziashahabi et al., 2020), and speech acts (Kasper, 1984; Koike, 1996; Cook and Liddicoat, 2002; Holtgraves, 2007). Research on the acquisition of L2 pragmatic comprehension shows that learners’ performance is related to three primary factors: within-implicature influence, learning experience and individual characteristics. However, learner belief—an important variable of individual difference characteristics—has not received much attention in L2 pragmatics. To address these gaps, this study investigates Chinese learners of English beliefs about developing L2 pragmatic comprehension from the perspective of the theory of planned behavior (TPB).

The TPB is one of the well-developed models for explaining, predicting, and changing human behavior in the field of social psychology (Ajzen, 1991, 2005). The theory has been applied to the study of a number of language learning behaviors and demonstrated excellent efficacy in explaining learners’ beliefs and intentions in various language learning environments (e.g., Lai, 2013; Zhong, 2013; Girardelli et al., 2017; Alhamami, 2018). Although TPB has potential in explaining engagement in language learning behavior, little research has analyzed L2 learners’ beliefs about and intention to develop pragmatic competence. Existing studies in the field of L2 pragmatics have examined whether comprehension is associated with the types of implicatures and learners’ learning experience (Roever, 2005; Taguchi, 2011). However, most studies have focused on the learners’ accuracy of comprehension, and studies that investigated what factors may influence learners’ pragmatic learning intention are scare.

Moreover, pragmatics has been largely neglected in formal classroom instruction in China, like in many other EFL contexts where the instruction focuses on grammar and vocabulary and the four skills (Taguchi and Roever, 2017). Investigation into learners’ beliefs about pragmatic competence would provide better insights into their actual pragmatic learning behavior. Such investigation will help describe how learners find an EFL context would facilitate or impede their pragmatic learning and would also shed light on the effective curriculum design.

Few studies have addressed the roles of learners’ internal characteristics in L2 pragmatic learning. Previous studies identified proficiency and cognitive processing abilities as two factors affecting pragmatic comprehension (Holtgraves, 2007; Taguchi, 2007, 2008a), but attempt to examine other internal characteristics variables, such as learner beliefs, has not often been made. Thus, this study aims to offer some insights into the extent to which L2 learners are able to comprehend English conversational implicatures. More importantly, it focuses on the analysis of the potential factors influencing their intentions to develop pragmatic comprehension and actual learning behavior in the Chinese EFL context. The adoption of TPB could extend our understanding of L2 pragmatic learning and the possible influencing factors from the perspective of social psychology.

## Literature Review

### Language Learner Beliefs

Research on language learner beliefs can be traced back to the mid-1980s pioneering empirical studies by Horwitz (1985) and Wenden (1986). Beliefs (or metacognitive knowledge, adopted from cognitive psychology) were defined as the ideas or opinions about aspects of L2 acquisition held by learners (Horwitz, 1987). Beliefs were viewed as cognitive in nature, stable and fallible (Wenden, 1991). More recently, learner beliefs have been regarded as one of individual difference characteristics, which have an effect on either the process or outcome of language learning (Kalaja and Barcelos, 2013; Kalaja et al., 2016). Beliefs were characterized as dynamic, complex and contradictory as well (Kalaja and Barcelos, 2003). As the research perspective on learner beliefs has shifted from an etic to an emic one (Negueruela-Azarola, 2011), the research methodology has changed accordingly, from relying on questionnaires to combining interviews with one or more instruments (Barcelos and Kalaja, 2011).

In this study, belief is considered as “the subjective probability of a relation between the object of the belief and some other object, value, concept, or attribute” (Fishbein and Ajzen, 1975, p. 131). Understanding how learners perceive their pragmatic comprehension and what factors influence their pragmatic learning intention and actual learning behavior can help language educators better support learners’ engagement in learning activities.

### Theory of Planned Behavior

The TPB (Ajzen, 1991, 2005) attempts to explain determinants of behavior. The theory asserts that behavior is intentional, and that human behavior is guided by the three types of beliefs: behavioral, normative, and control.

Behavioral beliefs are an individual’s beliefs about the likely consequences of a behavior (Fishbein and Ajzen, 1975; Ajzen, 2005). Behavioral beliefs are personal in nature and influence an individual’s attitude toward the behavior, i.e., positive or negative evaluations of performing a particular behavior (Ajzen, 1991, 2005). These evaluations have two dimensions: experiential attitude, individuals’ emotional response to the idea of performing the behavior, and instrumental attitude, determined by beliefs about the outcome of behavior.

Normative beliefs refer to beliefs influenced by the judgment and expectations of significant others (e.g., parents, spouse, friends, teachers, etc.) in our social and professional networks (Ajzen, 2005). Normative beliefs lead to perceived social pressures, individual’s perceptions of relevant others’ beliefs that he or she should or should not perform the behavior under consideration (Ajzen, 2005).

Control beliefs are an individual’s beliefs about the presence of factors that may facilitate or impede performance of the behavior (Ajzen, 2002). Control beliefs result in perceived behavioral control—the extent to which an individual believes one is capable of performing a behavior (Fishbein and Ajzen, 2010). Perceived behavioral control is related to two elements: self-efficacy and actual behavioral control. The former is concerned with individuals’ judgments of how well he or she can actually perform the behavior (Bandura, 1982), whereas the latter refers to the resources and factors available to a person which direct the intention into behavior (Ajzen, 1991).

The theory of planned behavior has successfully explained intentions and behaviors in a variety of language learning environments, including learners’ willingness to communicate in a target language community (Zhong, 2013), intentions to engage in foreign language learning (Alhamami, 2018), students’ in-class participation on an international branch campus (Girardelli et al., 2017) and self-directed use of technology for language learning (Lai, 2013). However, little research has explored its application in learners’ pragmatic competence. Therefore, this study aims to apply the TPB to explore learners’ beliefs about developing L2 pragmatic comprehension.

### Comprehension of Implicature

Comprehending implicatures can be a great challenge for L2 learners, because they need to decode both linguistic and contextual cues and to work out inferences of speakers’ implied intentions (Taguchi, 2013). Previous studies have found that learners are able to comprehend implied meaning, but their comprehension ability is significantly weaker than that of native speakers (Bouton’s, 1994; Roever, 2005, 2013; Taguchi, 2007, 2008a,^2011^).

Learners’ comprehension ability seems to be related to three primary factors: within-implicature influence, learning experience and individual characteristics. Within-implicature influence refers to the types of implicature and different comprehension loads caused by them (Taguchi and Bell, 2020). Bouton’s (1994) found that learners with over 17 months of residence in the U.S. achieved native-like comprehension accuracy for idiosyncratic implicature, but their comprehension of indirect criticism, sequence and Pope Question implicature (saying “Is the Pope catholic?” to mean something obvious) remained difficult due to the culture-specific nature of the formulaic implicatures, suggesting different levels of comprehension load among different implicature types. Similarly, Roever and his colleagues (Roever, 2005; Roever et al., 2014a) found that, for L2 learners, idiosyncratic implicature was significantly easier to understand than formulaic implicature. Taguchi showed that learners’ comprehension was faster and more accurate for indirect refusals than for indirect opinions (Taguchi, 2008a,^2011^) and over time learners gained in both accuracy and comprehension speed (Taguchi, 2007).

Learning experience encompasses learning contexts, target language contact and length of residence. Previous studies have mixed findings about the effect of learning context on implicature comprehension. Taguchi (2011) found the study abroad (SA) group achieved higher scores on the comprehension accuracy of routines than the at home (AH) group. However, Roever (2005) suggested that there is no significant effect of learning environment on learners’ implicature comprehension, as both SA and AH groups in his study performed similarly on comprehension of formulaic implicatures and idiosyncratic implicatures. Taguchi’s (2008b) study also showed that AH learners obtained a more profound gain than the SA group in accurate comprehension of indirect refusals, but they were similar in the comprehension of indirect opinions.

Yamanaka (2003) examined the effect of SA length on implicature comprehension. Results showed that long-term (more than 54 months) groups outperformed short-term (0–17 months) groups in comprehension scores. Taguchi (2008a) found that the amount of SA learners’ speaking and reading activities was correlated with their development in comprehension speed, but not in comprehension accuracy.

Learners’ individual characteristics, such as proficiency and cognitive processing abilities (Holtgraves, 2007; Taguchi, 2007, 2008a,b) also affect implicature comprehension. General proficiency has proved a strong indicator of implicature comprehension accuracy across studies (Cook and Liddicoat, 2002; Taguchi, 2011; Roever, 2013; Köylü, 2018). Moreover, cognitive variables, such as working memory and lexical access skills, were found to constrain or support implicature comprehension (Taguchi, 2008b).

As the above review indicates, existing studies have documented mixed findings even when L2 learners were exposed to similar learning environments (e.g., Yamanaka, 2003; Roever, 2013; Roever et al., 2014b). This suggests that other factors related to individual variation (e.g., learner beliefs) should be examined to develop a comprehensive understanding of pragmatic comprehension. Furthermore, little research has examined learners’ beliefs about pragmatic learning in the Chinese context. Therefore, a mixed-method approach was adopted to explore Chinese EFL learners’ pragmatic comprehension and their beliefs about L2 pragmatic learning. The present study addresses the following research questions:

(1) To what extent are the Chinese EFL learners able to comprehend English conversational implicatures?
(2) Which factors may influence their L2 pragmatic learning intention and learning behavior?

## This Study

### Participants

The study was conducted at a large public university in Southeast China. A group of 498 freshmen (192 males and 306 females; mean age 19.6) were selected based on the availability of the students and their willingness to participate. They were freshmen enrolled in the “College English Level 3” course. This course was offered to Chinese learners of English with an intermediate level of proficiency, as determined by a 2-h English placement test (measuring their listening and reading abilities) at the start of the academic year. The participants came from various regions in China. They were majoring in various disciplines, including law, biology, environmental science, engineering, journalism, business administration, etc. The participants had completed 10.5 years of formal English education on average. None of these students had lived in an English-speaking country for more than 1 month.

A subset of 12 students (six males and six females) were selected for follow-up interviews and stimulated recalls on the basis of the maximum variation sampling strategy (Dörnyei, 2007). This strategy allows us to examine the variation within the respondents and highlight any commonalities across the sampled diversity. In the web-based survey, all participants were required to decide whether to be interviewed subsequently or not, while only 21 students indicated their interest. Considering gender balance, age, academic majors, and the representation of different levels of pragmatic comprehension (i.e., high, mid and low scores collected from the web-based survey), this study finally selected 12 students to offer qualitative data.

### Instruments

Data were collected using a web-based survey, stimulated recall (SR) tasks and semi-structured interviews. The instruments are described as follows.

#### Web-Based Questionnaire

The data were collected *via* an online survey, consisting of multiple-choice listening questions (MCLQ) for comprehension of implicature, questions for demographic information and indication of to be interviewed. Following standard criteria for development of valid and reliable questionnaire (Dörnyei, 2003), the researcher started with a careful scrutiny of the relevant literature on conversational implicatures. Twelve items were adapted from previous studies (Roever, 2005; Roever et al., 2014a) to assess learners’ ability to interpret conversational implicatures. Each item contained a brief English description of the scenario, a two-turn dialogue, a question prompt and four response alternatives. The descriptions, dialogues and prompts were recorded by native English speakers in a clear voice. The audio-recorded conversations enabled participants to make their choices based on the speakers’ prosody. Next, the initial MCLQ was piloted with native English speakers to determine if the implicatures could be interpreted as intended. Two items that failed to gain consistent interpretations were eliminated. Finally, the 10-item MCLQ was given to three professors specializing in L2 pragmatics to obtain expert judgment of redundancy, content validity, readability and clarity of items (following Dörnyei, 2003). They all confirmed the validity of the instrument. Thus, the final version of the MCLQ contained 10 items (see Appendix A). The internal consistency reliability of the MCLQ was acceptable (α = 0.75).

#### Stimulated Recall Tasks

SR tasks were implemented to gain greater insights into learner beliefs about pragmatic learning. The task provided a concrete context for the elicitation of learner beliefs and helped participants retrieve and verbalize their decision-making processes. The stimuli used to activate participants’ memory structure in the study were an audio-recording of the MCLQ and its questionnaire responses. To enhance the validity of the qualitative responses, participants were asked to read the transcripts to validate or correct them if necessary, as suggested by Buss and Walter (2013). Moreover, following Mackey and Gass (2016), two raters coded all of the SR tasks. The researcher and one research assistant coded all transcripts of SR tasks, and this process yielded 96.67 percent inter-rater reliability.

#### Interviews

To complement the SR tasks, semi-structured interviews were conducted to probe for more information (Mackey and Gass, 2016). The interview questions (see Appendix B) were designed to capture the students’ self-evaluations of their current levels of English pragmatic comprehension, of their beliefs about pragmatic learning, and of their intentions to develop L2 pragmatic comprehension through the process of learning English. To increase the reliability of interview responses, as recommended by Mackey and Gass (2016), two raters coded the interview protocol. The researcher coded all transcripts of interviews and a second rater separately coded 30% of the transcripts. Then they reviewed the proportion of agreements and disagreements and found 92.18 percent inter-rater reliability. To reinforce the validity of the research, following Dörnyei (2007), the researcher also invited interview participants to discuss the findings of the research.

### Procedure

The web-based questionnaire data were collected to examine participants’ levels of pragmatic comprehension. First, participants received a link to the web-based questionnaires from their instructors after reading the Project Information Sheet (describing the study and its procedures) and signing a Consent Form during their “College English Level 3” listening classes. They completed the online survey on desktop computers (with earphones) in the language lab. They were given roughly 10 min to complete the survey without consulting a dictionary.

Next, after the quantitative data were preliminarily analyzed, a group of 12 students were selected for the subsequent SR tasks and semi-structured interviews. As the reliability of SR tasks is directly related to the time interval between the SR session and the event being discussed (Ren, 2014), the qualitative data were collected 2 days after participants’ completion of the MCLQ tasks.

Participants were recruited on a voluntary basis, and their participation would have no effect on their term grades. They were permitted to withdraw at any time. The SR tasks and interviews were conducted, on a one-on-one basis, in the participants’ L1 (Chinese) to make them less intimidating, and they were audio-recorded.

### Data Analysis

The scores of the 498 participants were imported into SPSS version 24.0 for analysis. Multiple-choice answers were coded “1” for the selection of the expected interpretation, and “0” for other options. This means that a score of 10 was the highest score the participants could achieve. The web-based survey allowed for the automatic scoring of items, which guaranteed the ease of administration and which also enhanced the test’s practicality (Taguchi and Roever, 2017). Descriptive analysis and a paired-sample *t*-test were conducted.

Both the SR protocols and interviews were fully recorded, immediately transcribed, coded and analyzed by the researcher and her research assistant, a Ph.D candidate in Applied Linguistics from the researcher’s college. The analysis of transcripts was informed by the four-step analysis approach developed by Holliday (2015), which involves coding, determining themes, constructing an argument and reviewing data.

Based on the TPB, we coded learners’ comments on their positive or negative evaluations of the likely consequences of pragmatic learning as behavioral beliefs; their perception about pressures from significant others to develop pragmatic competence as normative beliefs; their judgments of how well they can understand the intended meaning as self-efficacy beliefs; and their comments on whether certain resources under control for developing pragmatic comprehension as actual behavioral control. Once coding was completed, codes or recurring comments were grouped into themes. Frequencies were counted to identify the most frequently recurring themes. Next, the identified themes used as headings or subheadings and their representative extracts were used as evidence for points made in an argument. The processes of going back to the data, reassessing the codes and possibly refining the themes were added to support the argument.

## Results

### Comprehension of Conversational Implicature

Concerning the first research question, descriptive statistics and paired-sample *t*-test were conducted. As aforementioned, the MCLQ contained 10 items and participants could receive one point per item if they chose the desired answer. Thus, the full score for this task was 10 points. Among the 498 participants, 365 obtained six or more points. The result indicated that the learners were fairly successful at recognizing the implied meaning in the implicatures. It could be argued that the learners’ L2 pragmatic comprehension was in general at the upper intermediate level.

Table 1 presents the mean and standard deviation of the participants’ performance in each item. The data showed that almost every learner could infer what a speaker implied in his utterance that “I heard music from his room earlier” (Item 1), whereas they encountered most difficulties in interpreting a teacher’s indirect criticism of a student’s essay by commenting that “I thought it was well-typed” (Item 6).

**TABLE 1:** Participants’ performance in MCLQ (*n* = 498).

Item	Mean	Standard deviation
1	0.93	0.25
4	0.80	0.40
3	0.77	0.42
2	0.75	0.43
9	0.68	0.47
8	0.65	0.48
7	0.61	0.49
5	0.60	0.49
10	0.53	0.50
6	0.44	0.50

As presented in Table 2, the average score of the idiosyncratic implicatures (Items 1, 4, 5, 7, 8, and10) was 0.68, slightly higher than that of the formulaic implicatures (0.66). This indicated that for the Chinese EFL learners, the idiosyncratic implicatures was comparatively easier to decode than the formulaic implicatures. A paired-sample *t*-test showed that the learners’ interpretation of idiosyncratic implicatures were significantly higher than that of formulaic implicatures (*p* < 0.05), but the effect size was small (*d* = 0.11).

**TABLE 2:** Paired-sample *t*-test between the two types of implicatures (*n* = 498).

Comprehension scores	M	SD	*t(df)*	*p*(2-tailed)	*d*
Idiosyncratic implicature	0.68	0.23	1.97(496)	0.04	0.11
Formulaic implicature	0.66	0.29			

### Factors Influencing English as a Foreign Language Learners’ Pragmatic Learning Intention

Regarding the second research question, SR tasks and follow-up interviews were further conducted with 12 participants. To obtain an in-depth understanding of the Chinese EFL learners’ beliefs about L2 pragmatic learning, the researcher and her research assistant began coding learners’ beliefs that influenced their L2 pragmatic learning intention and actual learning behavior. The coded data verified several beliefs that motivated or demotivated students to develop pragmatic comprehension. The identified factors are listed in the following subsections in the order of importance, as indicated by the percentage of participants discussed the themes.

#### Importance of Second Language Pragmatic Comprehension

It was found that considerations of the importance of L2 pragmatic comprehension seemed to be related to behavioral beliefs (Ajzen, 1991, 2005). To recap, behavioral beliefs reflect an individual’s subjective beliefs about the consequences of a particular behavior and influence their attitudes toward performing a certain behavior. The interview data revealed that the learners held multi-layered, complex attitudes toward L2 pragmatic comprehension. They (91.67%) discussed the importance of acquiring the ability to understand the intended meaning from three aspects: (1) acquiring this ability is important but not urgent; (2) it is important for communication in the real world, but not for any particular academic purposes; and (3) it is important for some students but not all. The following are some typical responses on the importance of the L2 pragmatic comprehension. The excerpts are translated by the authors. All the names are pseudonyms.

(1) Important but not urgent

*I think understanding the deeper meanings of any utterance is very important; otherwise, what we can understand is only the literal meaning; in this case, mutual understanding cannot be achieved. In reality, I don’t think it is a very necessary skill for university students, as this ability has never been tested.* (Shiyuan)

(2) Important for communication but not for academic purposes

*From my previous experience, whenever I felt uncertain about or failed to understand my foreign friends’ mentality during a conversation, I easily became nervous, and then I cannot express myself clearly or accurately. So, I believe the ability to understand the intended meaning of the interlocutor is very important. Well, I have a ton of work to do, this ability has nothing to do with my academic performance*… (Zekun)

(3) Important for some students but not all

*This ability is important, especially for those who plan to study abroad, well, currently I don’t have such kind of plan, so I don’t really care about that*… *Moreover, to learn how to understand the implied meaning, I don’t think, is interesting*… (Fan)

As exemplified by the above responses, participants reported different, multi-layered opinions on the importance of acquiring L2 pragmatic comprehension, although they basically agreed that it was important. Ten out of the 12 participants (83.33%) explicitly expressed the view that they believed that developing the ability to comprehend implied meaning was critical but not urgent for EFL learners. Three participants (25%) claimed that this ability was important, but only for certain types of learners, such as those who planned to study abroad.

For the learners (Zekun) who found the ability to understand the intended meaning useful, their instrumental attitude (Fishbein and Ajzen, 2010) toward L2 pragmatic comprehension was favorable. In contrast, learners like Fan expressed her negative evaluation of developing L2 pragmatic comprehension, as she believed that learning how to understand the implied meaning was uninteresting and she did not have study abroad plan. Her response indicated that both her experimental attitude and instrumental attitude (Fishbein and Ajzen, 2010) were unfavorable. This finding is in line with the one reported by Härmälä et al. (2017), who found that students’ future study plan predicted their proficiency and opinion of English.

#### Perceived Self-Efficacy

Learners’ perceived self-efficacy can also influence their pragmatic learning intentions and actual behaviors. In this study, nine participants (75%) appeared to lack perceived confidence in their language proficiency. In other words, they perceived themselves as only having the competence to understand the intended meaning conveyed in simple English. This belief also impeded their pragmatic learning intention.

*I think only people who learn English very well need to have this ability. Simple dialogues like this, I can understand, but not too complicated ones, in which some big words or complex sentence patterns would be involved. Those are beyond my current proficiency level. And I feel this ability is too hard for me to develop*… (Boru)

While the low self-efficacy was related to deficiency in language proficiency, the high self-efficacy was found associated with their mastery of the L2 community’s customs and traditions. Ten participants (83.33%) attributed their success in recognizing speaker’s intention to their knowledge of the L2 culture. For instance, when asked to explain his choice for Item 3, Boru said that he believed that “being punctual” was one of the most important values that English speakers held. Boru’s response suggested that the mastery of L2 cultural norms along with reasoning ability facilitated the learners to derive conversational implicatures.

*I chose D for this item because I learned that being punctual is a commonly shared value in the English-speaking community. So, if students hand in their class projects late, they will get a lower grade, since they have broken the rule (being punctual). And in this scenario, the speaker cleverly replied “Do fish swim?” This looks like a question, but in fact it is an answer.* (Boru)

#### Lack of Opportunities and Resources

In the interviews, eight participants (66.67%) reported that some non-motivational factors such as availability of requisite opportunities and resources (e.g., the lack of exposure to the L2 community, limited time to engage in pragmatic learning and lack of instruction on L2 pragmatics) overshadowed the contribution of their intention to engage in pragmatic learning behavior. Five participants (41.67%) reported that they regarded the lack of exposure to the L2 community as an obstacle to their pragmatic learning.

*Interpreting the unsaid meaning would be extremely hard for people who had no first-hand experience. Learners can benefit from staying with the native speakers of the target language in their community. It would be very hard for speakers who have never been abroad to understand this. I’ve got a lot to handle*… *I have very limited time, so I won’t bother to learn it now.* (Boru)

As the above excerpt revealed, the EFL learners, such as Boru, still had few opportunities to communicate with native English speakers in real life, making it difficult for them to develop pragmatic comprehension.

According to the TPB (Ajzen, 1991, 2005), since the resources and opportunities available to a person dictate the likelihood of behavioral achievement, learners’ controlled ability can influence their L2 pragmatic-related intentions and behaviors *via* the belief that they simply have no control over the behavior. The following example indicated such a belief.

*I think this ability to decode the implied meaning is very useful for real-life communication, but my university does not offer instructions on this, so I think I am unable to learn knowledge about it. Right now, we still focus on exercises due to our exam-oriented education system, and I don’t think I have time to learn something else.* (Shengnan)

As Shengnan commented, due to the lack of explicit instruction on pragmatics, she had no intention to improve her L2 pragmatic competence. In addition, a few students reported that they were too busy to focus on pragmatic learning. The learners’ lack of intentions to engage in pragmatic learning was a joint result of low perceived behavioral control (e.g., exposure to the L2 community), low actual behavioral control (e.g., instruction offered) and time constrains.

#### Few Social Pressures on Developing Pragmatic Comprehension

The beliefs about undervalued pragmatic comprehension seemed to be associated with the learners’ normative beliefs (Ajzen, 2005). Three participants (25%) reported that they believed that pragmatic comprehension was undervalued by their instructors and universities as reflected by the fact that L2 pragmatic knowledge was rarely taught in class or tested in exams.

*I think this ability is quite useless for me. If teachers thought this ability was important to us, why not offer instructions on it? I would like to enlarge my vocabulary rather than learn something that won’t be tested at all.* (Minghao)

TPB posits that individuals who believe that important others want them to perform a certain behavior are more likely to do so (Ajzen, 1991). Thus, students whose teachers pressure them to learn L2 pragmatics knowledge or who would be tested about pragmatics would have greater intention to engage in L2 pragmatic learning themselves.

To sum up, the learners reported factors that might have affected their intention to develop pragmatic comprehension, including the usefulness of pragmatic comprehension in the EFL context, their perceived self-efficacy (e.g., language proficiency and perceptions of L2 culture), available opportunities and resources for learning (e.g., exposure to L2 community, time, instruction), and lack of social pressures (e.g., pragmatics is not tested in exams and undervalued by significant others).

## Discussion

This study examined Chinese EFL learners’ L2 pragmatic comprehension of conversational implicature and their beliefs about pragmatic learning by drawing on TPB (Ajzen, 1991, 2005). Concerning learners’ pragmatic comprehension, the quantitative results showed that approximately three quarters of the participants (365 out of 498) were rather successful in identifying intended meanings of conversational implicatures in the MCLQ, reflecting that their pragmatic comprehension was relatively high. This finding echoes previous studies on learners’ pragmatic comprehension, which found that pragmatic comprehension can be acquired in the foreign language learning environment (Taguchi, 2007; Ren, 2015).

The learners performed significantly better in idiosyncratic implicatures than in formulaic implicatures, indicating that formulaic implicatures may be more challenging for EFL learners. This finding corroborates with the conclusion of Roever et al. (2014a) that formulaic implicatures are more difficult than idiosyncratic implicatures for learners to comprehend. Unlike Bouton’s (1994, 1999) finding, the majority of the Chinese EFL learners were able to comprehend the two Pope Questions (Items 3 and 9). As Zufferey (2015) observed, learners from different cultural backgrounds differed in deriving the same implicatures. It is possible that Chinese EFL learners may know more about cultural-specific background knowledge of Pope Questions, which help them to comprehend this type of implicatures.

Findings also revealed that the difficulty levels of decoding the two types of implicatures are not clear-cut. The learners obtained higher scores for certain implicatures, including both idiosyncratic (e.g., Items 1 and 4) and formulaic implicatures (e.g., Items 2, 3 and 9), than others (Items 5, 7, 8, 9, and 10). A possible explanation is that the learners might have prior knowledge of the target language/culture and might have a higher degree of topic familiarity with some implicatures. However, the findings cannot be generalized to pragmatic comprehension in general, and more investigations are needed before a general conclusion can be drawn.

Regarding the second research question, the analyses of the SR tasks and interviews implied that a number of factors influenced the EFL learners’ beliefs about developing L2 pragmatic comprehension and may in turn have shaped their pragmatic learning behaviors, including learners’ behavioral beliefs about (learning) pragmatic comprehension, their perceived self-efficacy and actual behavioral control. The TPB claims that behavioral beliefs are personal in nature (Ajzen, 1991, 2005), which can help to explain the results that the learners held multi-layered, complex attitudes toward pragmatic comprehension and its acquisition. The result, to some extent, is consistent with findings reported by Borghetti and Beaven (2017), who found that students held contradictory attitudes and beliefs regarding language learning and use. Although most participants in the study held positive attitudes toward the ability to recognize the intended meanings in real-life communication, they tended to take negative attitudes toward L2 pragmatic learning. For example, one participant (Fan) commented that learning about L2 pragmatic features was tedious, and another participant (Shiyuan) reported that acquiring L2 pragmatic features would be useless for exam-oriented education, indicating that both their experiential and instrumental attitudes toward pragmatic learning were negative. As a result of these two attitudinal stances, it is reasonable to hypothesize that the learners would lack intentions to engage in pragmatic learning.

According to the TPB (Ajzen, 1991, 2005), self-efficacy beliefs or perceived behavior control are determined by the total set of accessible control determinants, including beliefs about the presence of factors that may facilitate or impede acquisition of pragmatic comprehension. In this study, the learners’ perceived behavioral control influenced their pragmatic-related behaviors *via* the belief whether they could perform certain behaviors (e.g., “Pragmatic comprehension is too hard for me to develop”) or whether they had control over certain behaviors (e.g., “My university does not offer instructions on L2 pragmatics, so I am unable to learn knowledge about it”). The results suggested that the learners’ perceived self-efficacy in pragmatic learning might relate to both linguistic factors (L2 proficiency) and socio-cultural factors (knowledge of L2 culture and norms). This finding echoed the previous results of the literature indicating that language learners’ low self-efficacy was associated with their low language proficiency (Truong and Wang, 2019; García-Gómez, 2020; Wang and Sun, 2020). Moreover, the current study revealed that the learners’ self-efficacy beliefs about pragmatic comprehension was also related to their knowledge about the L2 community’s tradition and customs. Learners who possessed knowledge of the target culture (e.g., being punctual) appeared to be more confident and successful in decoding the implied meaning.

The TPB posits that an individual’s intention to perform a behavior is influenced by their perceived behavioral control (Ajzen, 2005). If someone believes that they have control over the necessary resources and can engage in the behavior, they are more likely to act in that way. The interview data showed that the EFL learners to some degree found that they lacked control over the requisite opportunities and resources to develop their pragmatic comprehension in the EFL context. Consequently, few students would take actions to learn L2 pragmatic knowledge, although they generally believed that pragmatic comprehension was important for communication. The finding is consistent with the study by Yang and Ren (2019), in which EFL learners’ reluctance to develop their pragmatic awareness was attributed to few opportunities for practicing or applying their acquired L2 pragmatic knowledge.

The study has theoretical and pedagogical implications. At the theoretical level, the results corroborate the effectiveness of the TPB in explaining EFL learners’ beliefs about pragmatic comprehension. Thus, drawing on the TPB for this study confirms the possibility of employing different theoretical models from sociopsychology to examine learners’ motivation to learn a foreign language. Pedagogically, the use of TPB may help language teachers and test designers to understand learners’ beliefs about L2 pragmatic learning in the EFL context. Understanding the factors influencing learners’ intention will help to design more effective teaching curricula that may integrate pragmatic instruction and testing in the future. For example, technology-mediated instruction or feedback may be included in EFL contexts to afford learners authentic social contact (Gonzales, 2013; Sykes, 2013; González-Lloret, 2019).

## Conclusion

This study investigated Chinese EFL learners’ pragmatic comprehension of implicatures and their beliefs about pragmatic comprehension. The results demonstrated that the learners differed in comprehending conversational implicatures. In addition to implicature types, learner beliefs about pragmatic learning played a role in identifying the implied meanings. Specifically, attitudes toward pragmatic comprehension, the perceived self-efficacy about language proficiency and L2 cultural knowledge, the actual behavior control over the opportunities and resources for pragmatic learning, and the perceptions of lack of social pressures together influenced their intention to develop pragmatic comprehension.

Future research is needed to explore how learner belief may influence pragmatic learning of other aspects such as pragmatic production. In addition, given that the present study only investigated Chinese EFL learners, future studies need to expand the research scope to include learners from other L1 backgrounds, as learners’ derivations of implicatures are closely related to their cultural backgrounds.

## Data Availability Statement

The original contributions presented in the study are included in the article, further inquiries can be directed to the corresponding author.

## Ethics Statement

The studies involving human participants were reviewed and approved by Xiamen University. The patients/participants provided their written informed consent to participate in this study.

## Author Contributions

The author confirms being the sole contributor of this work and has approved it for publication.

## Conflict of Interest

The author declares that the research was conducted in the absence of any commercial or financial relationships that could be construed as a potential conflict of interest.

## Publisher’s Note

All claims expressed in this article are solely those of the authors and do not necessarily represent those of their affiliated organizations, or those of the publisher, the editors and the reviewers. Any product that may be evaluated in this article, or claim that may be made by its manufacturer, is not guaranteed or endorsed by the publisher.

## Appendix A

### Multiple Choice Questions

1. Jack is talking to his housemate Sarah about another housemate, Frank.
  Jack, “Do you know where Frank is, Sarah?”
  Sarah, “Well, I heard music from his room earlier.”
  *What does Sarah probably mean?*
  A. Frank forgot to turn the music off.
  B. Frank’s loud music bothers Sarah.
  C. Frank is probably in his room.
  D. Sarah doesn’t know where Frank is.

2. Toby and Ally are trying a new restaurant in town. Toby is eating something but Ally can’t decide what to have next.
  Ally, “How do you like what you’re eating?”
  Toby, “Well, let’s just say it’s colorful.”
  *What does Toby probably mean?*
  A. He thinks it is important for food to look appetizing.
  B. He thinks food should not contain artificial colors.
  C. He wants Ally to try something colorful.
  D. He does not like his food much.

3. Maria and Frank are working on a class project together but they won’t be able to finish it by the deadline.
  Maria, “Do you think Dr. Gibson is going to lower our grade if we hand it in late?”
  Frank, “Do fish swim?”
  *What does Frank probably mean?*
  A. He thinks they should change the topic of their projects.
  B. He thinks their grades will not be affected.
  C. He did not understand Maria’s question.
  D. He thinks they will get a lower grade.

4. Jane notices that he co-worker Sam is dirty all over and has holes in his pants and scratches on his face and hands.
  Jane, “What happened to you?”
  Sam, “I rode my bike to work.”
  *What does Sam probably mean?*
  A. Today he finally got some exercise biking.
  B. He hurt himself biking.
  C. It’s hard to get to work without a car.
  D. He enjoys biking.

5. Felicity is talking to her co-worker Brian during a coffee break.
  Felicity, “So, life must be good for you. I hear you got a nice raise.”
  Brian, “This coffee is awfully thin. You’d think they’d at least give us decent coffee.”
  *What does Brian probably mean?*
  A. He does not want to talk about how much money he makes.
  B. He likes his coffee strong.
  C. He is planning to complain about the coffee.
  D. He doesn’t care very much about money.

6. Jose and Tanya are professors at a college. They are talking about a student, Derek.
  Jose, “How did you like Derek’s essay?”
  Tanya, “I thought it was well-typed.”
  *What does Tanya probably mean?*
  A. She did not like Derek’s essay.
  B. She likes it if students hand in their work type-written.
  C. She thought the topic Derek had chosen was interesting.
  D. She doesn’t really remember Derek’s essay.

7. Carrie is a cashier in a grocery store. After work, she’s talking to her friend Simon.
  Carrie, “I guess I’m getting old and ugly.”
  Simon, “What makes you say that?”
  Carrie, “The men are beginning to count their change.”
  *What does Carrie probably mean?*
  A. She has given wrong change a number of times, so people count their change now.
  B. Male customers aren’t admiring her anymore like they used to.
  C. The store might lose business if she doesn’t look good.
  D. It gets harder to give correct change as you get older.

8. Hilda is looking for a new job. She’s having lunch with her friend John.
  John, “So how’s job search coming along?”
  Hilda, “This curry is really good, don’t you think?”
  *What does Hilda probably mean?*
  A. She’s very close to finding a job.
  B. She’ no longer looking for a job.
  C. She just found a job.
  D. Her job search isn’t going very well.

9. Mike is trying to find an apartment in New York City. He just looked at a place and is telling his friend Jane about it.
  Jane, “Is the rent high?”
  Mike, “Is the Pope Catholic?”
  *What does Mike probably mean?*
  A. He doesn’t want to talk about the rent.
  B. The rent is high.
  C. The apartment is owned by the church.
  D. The rent isn’t very high.

10. At a recent party, there was a lot of singing and piano playing. At one point, Matt played the piano while Brian sang. Jill was not at the party, but her friend Linda was.
  Jill, “What did Brian sing?”
  Linda, “I don’t know what he thought he was singing, Matt was playing ‘Yesterday’.”
  *What does Linda probably mean?*
  A. Brian sang very badly.
  B. She was only interested in Matt and didn’t listen to Brian.
  C. Brian and Matt were not doing the same song.
  D. The song that Brian sang was “Yesterday.”

## Appendix B

### Interview Questions

1. As a university student, what do you expect to achieve from learning English?
2. How do you evaluate your current ability to decode the implied meaning in English?
3. Do you think the ability to understand the unsaid meaning is important? Why?
4. What made you believe that ability to interpret intended meanings behind the utterance in English is important or not?
5. What could be the factors facilitating or impeding your improvement of the aforementioned ability?
6. What efforts have you made or would you like to make to improve the aforementioned ability while learning English? Why have you made or would like to make these efforts?
